# Study on expelled but viable zooxanthellae from giant clams, with an emphasis on their potential as subsequent symbiont sources

**DOI:** 10.1371/journal.pone.0220141

**Published:** 2019-07-19

**Authors:** Shin-Ya Morishima, Hiroshi Yamashita, Shizuka O-hara, Yuji Nakamura, Vanessa ZhiQin Quek, Momo Yamauchi, Kazuhiko Koike

**Affiliations:** 1 Graduate School of Integrated Sciences for Life, Hiroshima University, Higashi-Hiroshima, Hiroshima, Japan; 2 Research Center for Subtropical Fisheries, Seikai National Fisheries Research Institute, Fisheries Research and Education Agency, Ishigaki, Okinawa, Japan; 3 Okinawa Prefectural Fisheries Research and Extension Center, Ishigaki, Okinawa, Japan; 4 Department of Biological Science, National University of Singapore, Singapore, Singapore; 5 School of Applied Biological Science, Hiroshima University, Higashi-Hiroshima, Hiroshima, Japan; Evergreen State College, UNITED STATES

## Abstract

Unlike most bivalve shellfishes, giant clams (tridacnines) harbor symbiotic microalgae (zooxanthellae) in their fleshy bodies. Zooxanthellae are not maternally inherited by tridacnine offspring, hence, the larvae must acquire zooxanthellae from external sources, although such algal populations or sources in the environment are currently unknown. It is well known that giant clams expel fecal pellets that contain viable zooxanthellae cells, but whether these cells are infectious or just an expelled overpopulation from the giant clams has not been investigated. In this study, we observed the ultrastructural and photosynthetic competencies of zooxanthellae in the fecal pellets of *Tridacna crocea* and further tested the ability of these cells to infect *T*. *squamosa* juveniles. The ultrastructure of the zooxanthellae cells showed that the cells were intact and had not undergone digestion. Additionally, these zooxanthellae cells showed a maximum quantum yield of photosystem II (*Fv/Fm*) as high as those retained in the mantle of the giant clam. Under the assumption that feces might provide symbionts to the larvae of other giant clams, fecal pellets from *Tridacna squamosa* and *T*. *crocea* were given to artificially hatched 1-day-old *T*. *squamosa* larvae. On the 9^th^ day, 15–34% of the larvae provided with the fecal pellets took up zooxanthellae in their stomach, and on the 14^th^ day, zooxanthellae cells reached the larval margin, indicating the establishment of symbiosis. The rate reaching this stage was highest, ca. 5.3%, in the larvae given whole (nonhomogenized) pellets from *T*. *crocea*. The composition of zooxanthellae genera contained in the larvae were similar to those in the fecal pellets, although the abundance ratios were significantly different. This study is the first to demonstrate the potential of giant clam fecal pellets as symbiont vectors to giant clam larvae. These results also demonstrate the possibility that fecal pellets are a source of zooxanthellae in coral reefs.

## Introduction

In tropical and subtropical seas, coral-algal symbiosis is the foundation of coral reef ecosystems. The algal symbionts, specifically dinoflagellates in the family Symbiodiniaceae (commonly called “zooxanthellae”), consist of genetically diverse groups traditionally referred to as clades A to I [1], and some of the clades have been formally described as genera recently [2]. Zooxanthellae are well known as coral symbionts; however, they can be found within various taxonomic groups. For example, some of the members of Anthozoa, Scyphozoa, Hydrozoa, Gastropoda, and Bivalvia, in addition to some sponge, foraminifera, flatworm, and ciliate species, harbor zooxanthellae (see [3] for review; [4]). Most of these host species retain zooxanthellae cells intracellularly; however, Bivalvia hosts, as represented by tridacnid shellfishes (giant clams), retain zooxanthellae cells in a specialized tubular system that extends from the stomach, which is referred to as zooxanthellal tubes [5]. Giant clams are important fishery resources, as they are used as food, as aquarium pets and for ornamental purposes. Currently, 13 extant species are recognized [6], and three species are categorized within the Vulnerable A2cd category on the International Union of Conservation of Nature (IUCN) Red List of Threatened Species [7]. Therefore, giant clam aquaculture has been practiced in many countries [8, 9, 10]. Giant clams are highly dependent on assimilation by photosymbionts for their nutritional requirements (e.g., glucose and amino acids [11]), with zooxanthellal photosynthates providing more than 50% of such nutrients [12, 13]. However, some reports suggest that they might also utilize nutritional resources provided by algae ingested as food during filter feeding [14, 15]. Three genetically distinct zooxanthellae clades or genera, namely, clade A = genus *Symbiodinium*, clade C = genus *Cladocopium*, and clade D = genus *Durusdinium* have been detected from giant clams in the Pacific Ocean [16–19]. The compositions of these zooxanthellae genera can differ among environments [17], geographical locality [19], clam species, and/or growth stages [18, 19].

Zooxanthellae are essential for host species. However, only a few host species inherit zooxanthellal cells from their parents. This vertical transmission system can be found within Acoelomorph flatworms [4], some scleractinian species [20, 21], and some members of Hydrozoa/Octocorallia/Actiniaria [22]. In other words, most of the host species must acquire their own zooxanthellae from surrounding environments (horizontal transmission). Thus, the zooxanthella population in an environment is essential for most host animals, and the genetic diversity of zooxanthellae in an environment [23–28] and their actual abundances [27, 29] have been reported. Corals can discharge their zooxanthellae into surrounding environments even under nonstressful conditions [30], and these zooxanthellae are considered to accumulate in the environment [27]. However, most of the discharged zooxanthellae cells were degraded or damaged [31] and were probably noninfectious. Thus, it is unlikely that the zooxanthellae released from corals become possible symbiont sources for the symbiont-bearing species that must acquire zooxanthellae horizontally. On the other hand, fecal pellets from tridacnine clams, such as *Tridacna maxima*, *Tridacna gigas*, and *Tridacna derasa*, have been found to contain numerous intact zooxanthellae cells [12, 14, 32]. Additionally, [12] reported that the zooxanthellae population in *T*. *maxima* fecal pellets was photosynthetically active and showed a carbon fixation rate of 1.9–4.0 mg C (mg Chl *a*)^-1^ h^-1^. The same authors showed that fecal pellets collected from *T*. *maxima*, *T*. *crocea*, *T*. *squamosa*, and *Hippopus hippopus* produced viable cultures of zooxanthellae in growth media. These results suggest that tridacnine clams can supply a large number of healthy zooxanthellae cells to coral reef environments via their fecal pellets. If such zooxanthellae have the potential to be infectious, one of the probable zooxanthellae sources in coral reefs would be identified. In the present study, to connect the missing link in the symbiont cycle in coral reef ecosystems, the basic properties of fecal pellets were initially investigated to evaluate the possibility of fecal pellets being symbiont vectors, followed by an aquarium experiment that demonstrated the ability of zooxanthellae in fecal pellets to infect aposymbiotic giant clam larvae.

## Materials and methods

### Observation of zooxanthellae in fecal pellets

Six individuals of *T*. *crocea*, those certified to be legally caught in the Okinawa region, Japan, were purchased from an aquarium shop. They were reared in a 50 L aquarium with artificial seawater (LIVESea Salt, Delphis, Hyogo, Japan) and under illumination (photon flux density (PFD) = ca. 400 μmol photons m^-2^ s^-1^ where the giant clams were located) which was provided by a metal halide lamp (Crystal Neo Halide 150, GEX, Osaka, Japan). The water temperature was maintained at approximately 28 °C by a chiller (Zc-100α, Zensui, Osaka, Japan).

**Fig 1:**
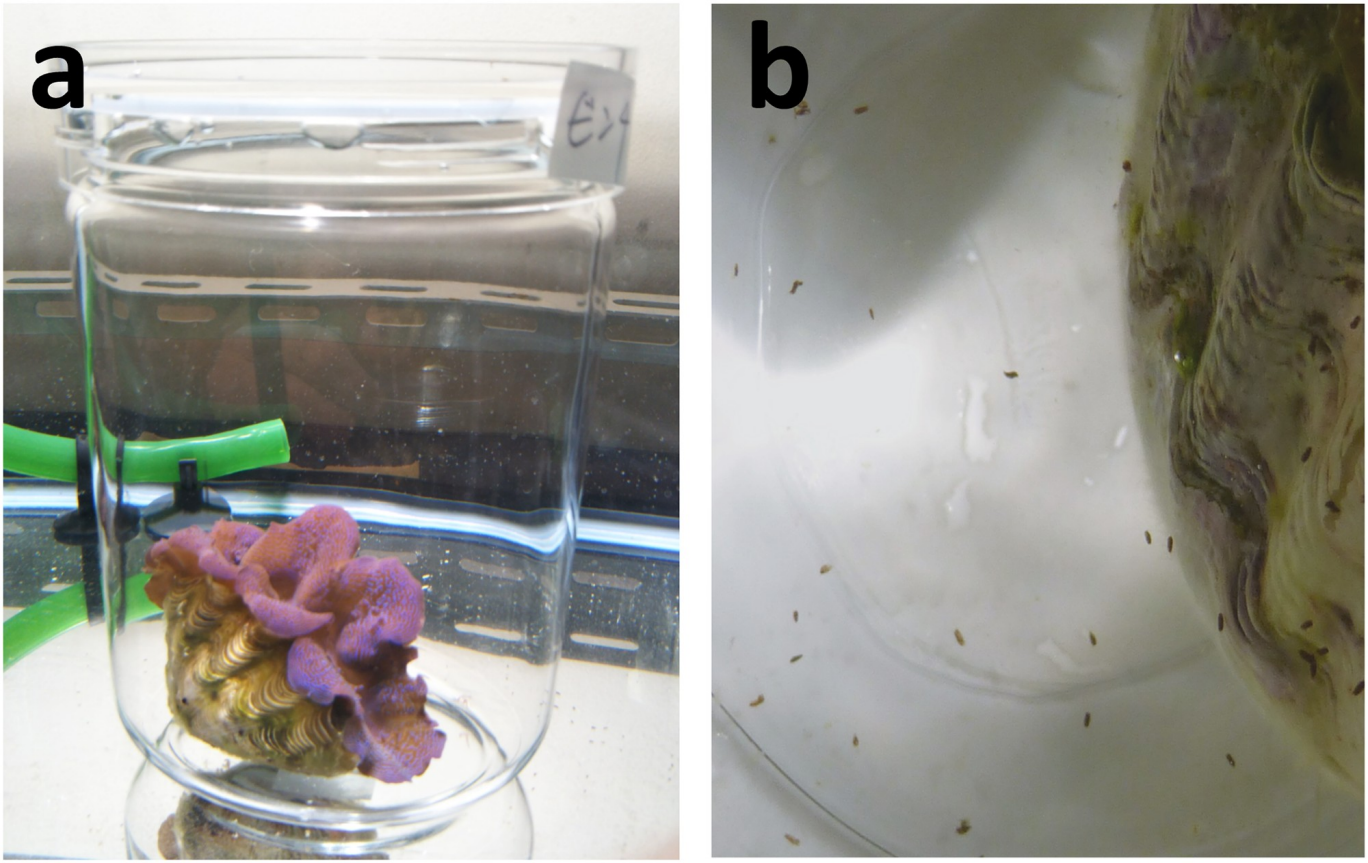
Procedure used to collect fecal pellets in the aquarium experiment. (a) Incubation of *Tridacna crocea* in a glass jar. (b) The fecal pellets (brown dots) expelled by *T*. *crocea*.

To collect fecal pellets, six individuals were separately placed in 1.3 L glass jars (98 mm Φ × 168 mm H) filled with artificial seawater and submerged in the aquarium water (Fig 1a). After 5 h of incubation (10 am—3 pm), the giant clams were removed, and fecal pellets were concentrated with reverse filtration by removing the water via a PVC (89 mm Φ) cylinder that was inserted into the jar with 20 μm nylon mesh attached at one end. After incubation, numerous large fecal pellets that had accumulated at the bottom of the jar were visually observed to have a rigid texture (Fig 1b). To determine the status of the algae contained in the fecal pellets, they were observed under a fluorescence microscope (IX 71, Olympus, Tokyo, Japan) under blue light excitation. Morphological observations were also performed by transmission electron microscopy (TEM); The fecal samples collected from one of the individuals were fixed and treated according to the method of [4], and ultrathin sections were observed under both a light microscope and TEM (JEM-1200EX, JEOL, Tokyo, Japan).

To determine the photosynthetic competency, the maximum quantum yield of photosystem II (*Fv/Fm*) of the zooxanthellae in the pellets was measured using a microscopy-type pulse amplitude-modulated (PAM) fluorometer (Micro-FluorCam FC 2000, Photon Systems Instruments, Brno, Czech Republic) at the single-cell level. A portion of the concentrated seawater that contained fecal pellets was transferred to a dish, and zooxanthellae cells were suspended in filtered seawater by homogenizing the fecal pellets with a hand pestle or vigorous pipetting. The measurements were performed separately for all six individuals. To compare the *Fv/Fm* values of these zooxanthellae with those retained in the mantle tissue, a piece of marginal mantle tissue (ca. 0.5 mm × 0.5 mm) was excised with small scissors and tweezers from those six individuals and transferred to a 1.5 ml microtube. The zooxanthellae in the tissue pieces were also extracted with the hand pestle. All samples were kept under dark conditions for more than 30 minutes to relax photosystem II, and then the *Fv/Fm* value was measured with the microscopy-type PAM fluorometer following the protocol of [31]. A total of 40 ~ 120 cells were subjected to this measurement.

### Fecal pellet expulsion under light and dark conditions

Three giant clams were individually placed in 5 L glass beakers filled with artificial seawater, which were placed in an air-conditioned room, where the temperature was set to 27 °C under illumination (PFD = 150 μmol photons m^-2^ s^-1^) provided by a white LED light (Clear LED SG 600 W, GEX). The light-dark cycle was 12 h:12 h. The seawater was thoroughly changed twice a day at 9 am and 6 pm, and all the fecal pellets that had accumulated on the bottom of the beaker were retrieved by a pipet at the same time. This process was continued for a total of 3 days. To determine the fecal pellet expulsion under dark conditions, the same beakers with giant clams that were previously exposed under a light-dark cycle for 3 days were covered with a shade curtain for 2 days for acclimation and kept under dark conditions for an additional 3 days. During these additional 3 days, all the fecal pellets were retrieved twice a day according to the abovementioned method. The numbers of expelled fecal pellets in each sample collected every 3 days under the light-dark cycle conditions and 3 days under the dark conditions were separately counted without the aid of a microscope. Additionally, approximately 20% of the pellets were randomly selected, and their images under a binocular (SZX10, Olympus, Tokyo, Japan) were captured with a camera (DP20, Olympus). The volumes of the captured fecal pellets were estimated with ImageJ 1.52a [33] according to the following method. The pellets within a photograph were initially converted to 8-bit images and selected while discriminating the background. These images were then transformed to ellipses, and their volumes were determined based on the longitudinal and latitudinal diameters.

### Infection experiment

#### Larva and zooxanthella sources

To test the hypothesis that the zooxanthellae in the fecal pellets can form a symbiosis with giant clam larvae, artificially hatched veliger larvae of *T*. *squamosa* at 24 h after fertilization were obtained from the Okinawa Prefectural Fisheries Research and Extension Center Ishigaki Branch (OPFREC), Okinawa, Japan. Approximately 9,000 individuals were randomly distributed to 18 beakers (approximately 500 individuals per beaker), each filled with 3 L filtered seawater (1 μm filtrate).

Five zooxanthellal sources were added to the beakers in triplicate, namely, source 1: freshly isolated zooxanthellae from adult *T*. *squamosa* mantle (FIZ hereafter), source 2: homogenized fecal pellets of *T*. *squamosa* [HF (Ts) hereafter], source 3: unhomogenized and intact whole fecal pellets of *T*. *squamosa* [WF (Ts) hereafter], source 4: homogenized fecal pellets of *T*. *crocea* [HF (Tc) hereafter], and source 5: intact whole fecal pellets of *T*. *crocea* [WF (Tc) hereafter]. The remaining three beakers served as controls without any zooxanthellae but with filtered seawater only (SW, hereafter). Zooxanthellae were supplied every 2 days at the time of water exchange (see the following section).

For FIZ, zooxanthellae cells were extracted from at least three adults of *T*. *squamosa* (shell length 50.0–58.3 mm) according to [34]. Briefly, all tissue of *T*. *squamosa* was transferred to a blender with seawater, homogenized several times for ten seconds, and then the homogenate was passed through a mesh strainer (ca 0.5 mm mesh) to remove the animal tissues. Finally, the zooxanthellae cells in the filtrates were quantified under a microscope. The cells were added to the beakers containing *T*. *squamosa* larvae at a final concentration of 30 cells ml ^-1^. This given cell density was determined experientially by [34] and applied to the following zooxanthellal sources. For HF (Ts) and WF (Ts), a total of 10 adult *T*. *squamosa* individuals (shell length: 50 mm—137 mm) stocked in the OPFREC were placed in a 150 L outdoor tank supplied with running seawater, and the fecal pellets deposited after 4 h were retrieved with a pipet from the tank bottom. To determine the zooxanthella cell density in a single pellet, a total of 10 medium-sized pellets (longitudinal length = ca. 1.5 ~ 2 mm) were selected from the stock of the abovementioned fecal pellets and homogenized with a hand pestle and/or vigorous pipetting in a known volume of filtered seawater. Thus, the suspended zooxanthellae cells were counted using a hemocytometer under a microscope to determine the approximate cell density (average) per single fecal pellet. Based on this cell density, the fecal pellets equivalent to a final concentration of 30 cells ml^-1^ were gently homogenized with a hand pestle and/or vigorous pipetting while trying not to destroy the zooxanthellae cells and supplied as HF (Ts). Those that were not homogenized (= whole intact pellets) represented WF (Ts). For HF (Tc) and WF (Tc), the same procedure used for HF (Ts) and WF (Ts) was followed, but the pellets were obtained from a total of 5 adult *T*. *crocea* individuals (shell length 62 mm—67.8 mm).

Unused FIZ and fecal pellets were centrifuged to remove the seawater supernatant and kept in a freezer for further zooxanthella genus analysis (see below section). To determine the physiological competency of the zooxanthellal sources in FIZ, HF (Ts), and HF (Tc), cellular *Fv/Fm* values were measured for an average of 40 cells (14 ~ 75 cells) using microscopy-type PAM following the abovementioned protocol.

#### Incubation

All the beakers were covered with wrapping films and submerged in a 150 L tank supplied with overflowing seawater to maintain the ambient water temperature. This tank was placed in a semienclosed outdoor cabin where natural sunlight was provided through a transparent PVC roof. The air PFD during the experimental period did not exceed 1500 μmol photons m^-2^ s^-1^. The temperature range of the running seawater during the experimental period was 24 °C to 27 °C. The water in the beakers was changed every two days by removing the water with a PVC cylinder with 20 μm mesh fixed to its open end to prevent removal of the larvae. After filling the beakers with new filtered seawater up to the 3 L level, the five abovementioned zooxanthellal sources were again added. This procedure was repeated 6 times until the 12th day of the experiment.

#### Observation of larvae

Because repeated sampling might cause unwanted damage to the larvae, the uptake of zooxanthellae cells and symbiosis establishment in the larvae were observed preliminarily on the 9th day after fertilization and finally on the 14th day. In giant clam seed production, the 12th day has been empirically recognized as the boundary between unsuccessful infection and symbiosis establishment [10]. On the 9th day, approximately 100 ml of water was carefully obtained from all the beakers to collect the larvae, and 10 ~ 62 individuals were observed under a fluorescence microscope and returned to the beakers. On the 14th day, all larvae were collected on a 20 μm mesh screen and rinsed with filtered seawater to remove free zooxanthella cells, and 102 ~ 135 individuals from each beaker were observed under a fluorescence microscope (CKX-41, Olympus) with blue light excitation. The rates at which the larvae took up the cells in their stomachs (uptake stage) and at which the cells reached the margin (symbiosis stage) were separately determined [15]. Larvae under the transient period when the zooxanthellae cells were transported from a stomach to the mantle edge and the zooxanthella cells extended from a stomach and to the edge were identified as the uptake stage. After these observations, all the larvae were kept in a freezer for further identification of Symbiodiniaceae genera.

#### Zooxanthella identification for the fecal pellets and infected larvae

The frozen fecal samples were thawed at room temperature and washed with ultra-pure water three times by centrifugation. Then, 100 μl TE buffer was added and subjected to bead-beating homogenization for 3 minutes using 0.5 mm micro glass beads and a bead-beater (Mini-BeadBeater-8, Biospec Products, Bartlesville, OK, USA) at 3,200 rpm to extract total DNA [30]. The frozen larval samples were also thawed at room temperature and subjected to the phenol/chloroform method using CHAOS solution (4 M guanidine thiocyanate, 0.1% N-lauroyl sarcosine sodium, 10 mM Tris pH 8.0, 0.1 M 2-mercaptoethanol) following the protocol in [35].

The extracted DNA samples were subjected to quantification of zooxanthella genera. It has been shown that giant clams exclusively harbor clades A (*Symbiodinium*), C (*Cladocopium*) and D (*Durusdinium*); therefore, we targeted these three genera using specific primer sets [30]. A quantitative PCR (qPCR) assay was employed following the protocol in [30], namely, using the genus-specific primer pairs of SymA28S-1F and SymA28S-1R for *Symbiodinium*, SymC28S-1F and SymC28S-1R for *Cladocopium*, and SymD28S-1F and SymD28S-1R for *Durusdinium*. The analyses were conducted with a StepOne qPCR thermal cycler (Applied Biosystems, Foster City, CA, USA) and TB Green chemistry (TaKaRa TB Premix Ex Taq II kit, Takara Bio Inc., Shiga, Japan). The qPCR results were expressed as zooxanthella cell numbers for each genus and their relative percentages by referring to standard curves (Ct vs cell number) derived from the known cell numbers of culture strains CS-161 (*Symbiodinium*; purchased from the Commonwealth Scientific & Industrial Research Organization, Australia), CCMP2466 and CCMP2556 (*Cladocopium* and *Durusdinium*, respectively; purchased from the Provasoli-Guillard National Center for Culture of Marine Phytoplankton, USA).

### Statistical analysis

All statistical analyses were performed using R version 3. 3. 3 [36]. Student’s t-test was used to compare the mean *Fv/Fm* values of zooxanthella cells in mantles and fecal pellets. The test was performed separately for each of the six individuals. The null hypothesis was that there was no difference in the mean between the two sample types. A paired t-test was performed to determine the differences in the fecal pellet expulsion rate under light and dark conditions. The null hypothesis was that there was no difference between dark and light conditions. For both t-tests, the null hypothesis was rejected when the *p*-value was below 5.0%. A one-way analysis of variance (ANOVA) was used to determine the difference among all five zooxanthellal source groups excluding the control in terms of the rates of larval uptake and symbiosis establishment. The null hypothesis was that there were no differences among the zooxanthellal sources. If the null hypothesis was rejected for the larval analyses, a Tukey HSD test was performed to compare the multiple groups. A chi-squared test was performed to compare the genus compositions of the added zooxanthellal sources and the larvae reared with the different zooxanthellal sources. The null hypothesis was that there was an association between the zooxanthellal sources and the zooxanthellal community hosted by the larvae. The same test was applied for the *Fv/Fm* value. The null hypothesis was that there were associations among the zooxanthellal sources. In all tests, a significant *p*-value was set to 5.0%.

## Results

### Observation of zooxanthellae in fecal pellets

**Fig 2:**
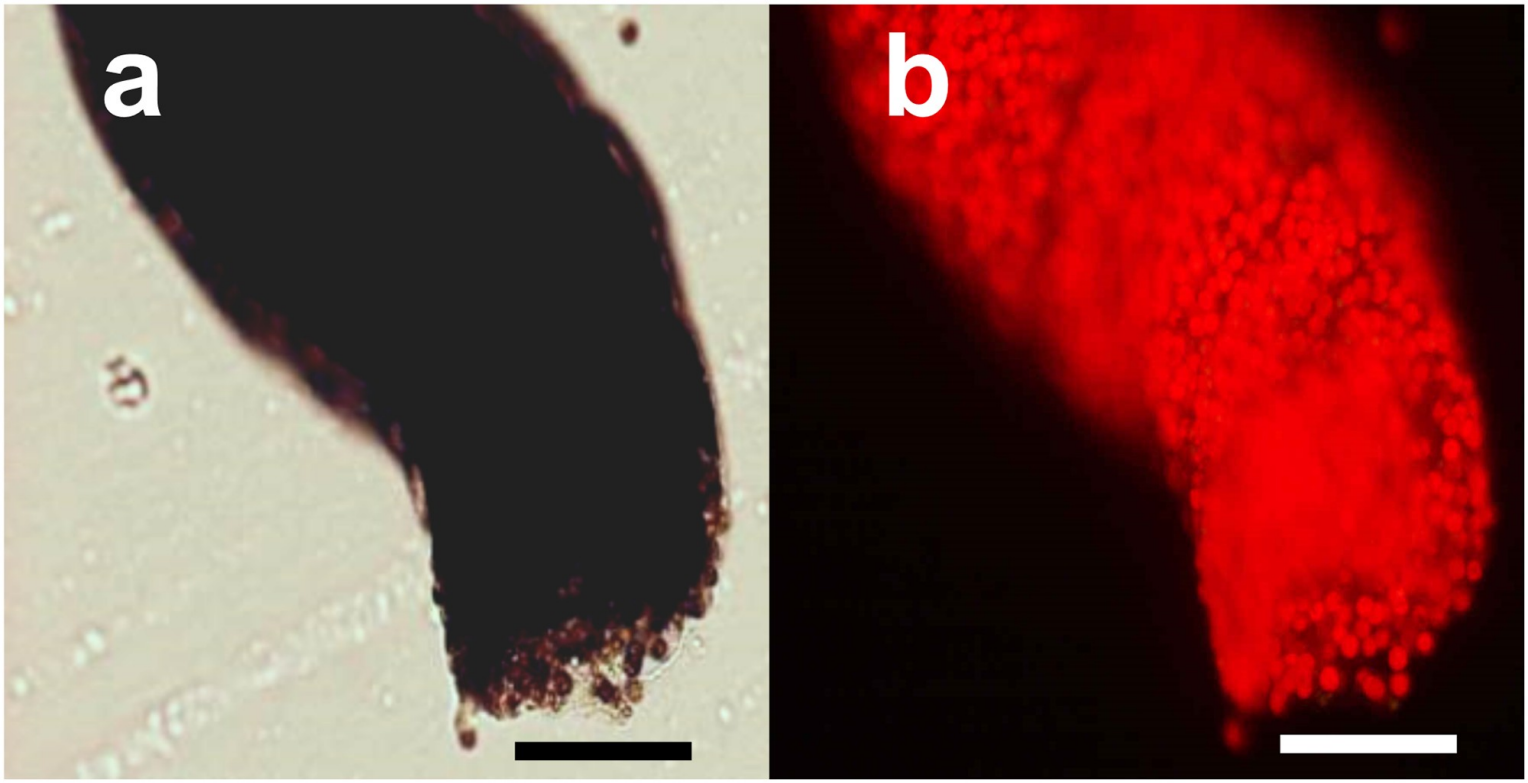
Light micrographs of a fecal pellet expelled from *Tridacna crocea*. (a) Transparent image and (b) fluorescent image under blue light excitation of a fecal pellet. Scale bars = 100 μm.

**Fig 3:**
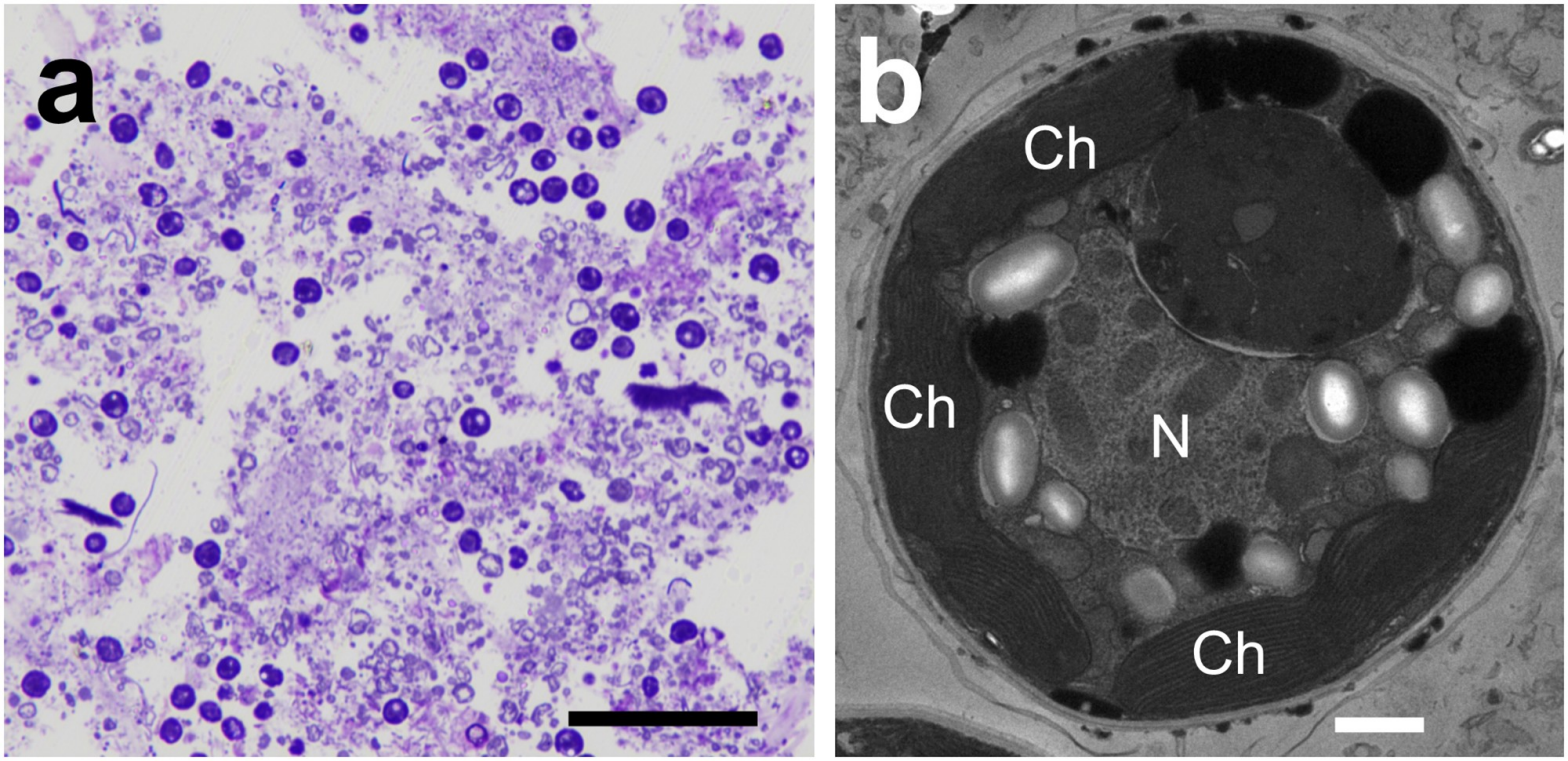
Micrographs of thin sections of a fecal pellet expelled from *Tridacna crocea*. (a) A light micrograph of a fecal pellet section showing numerous particles stained with toluidine blue, namely, zooxanthella cells. (b) A transmission electron micrograph of a zooxanthellal cell in a fecal pellet. Scale bar in the light micrographs = 50 μm, and that in the transmission electron micrograph = 1 μm. N, nuclei; Ch, chloroplast.

After 5 h of incubation in the jars, fecal pellets accumulated at the bottom of the jar (Fig 1). Light and fluorescence micrographs of the fecal pellet are shown in Fig 2. Under blue light excitation, numerous bright chlorophyll autofluorescent granules, namely, zooxanthella cells, were observed (Fig 2b). In the thin sections of the fecal pellets (Fig 3a), toluidine blue-stained granules of the zooxanthellae scattered across the transverse fecal section were observed under light microscopy, and the zooxanthella cells in the pellet showed intact intracellular structures under TEM (Fig 3b). Chloroplast thylakoid maintained an intact structure (Fig 3b, Ch), insisting that the cells were photosynthetically competent.

**Fig 4:**
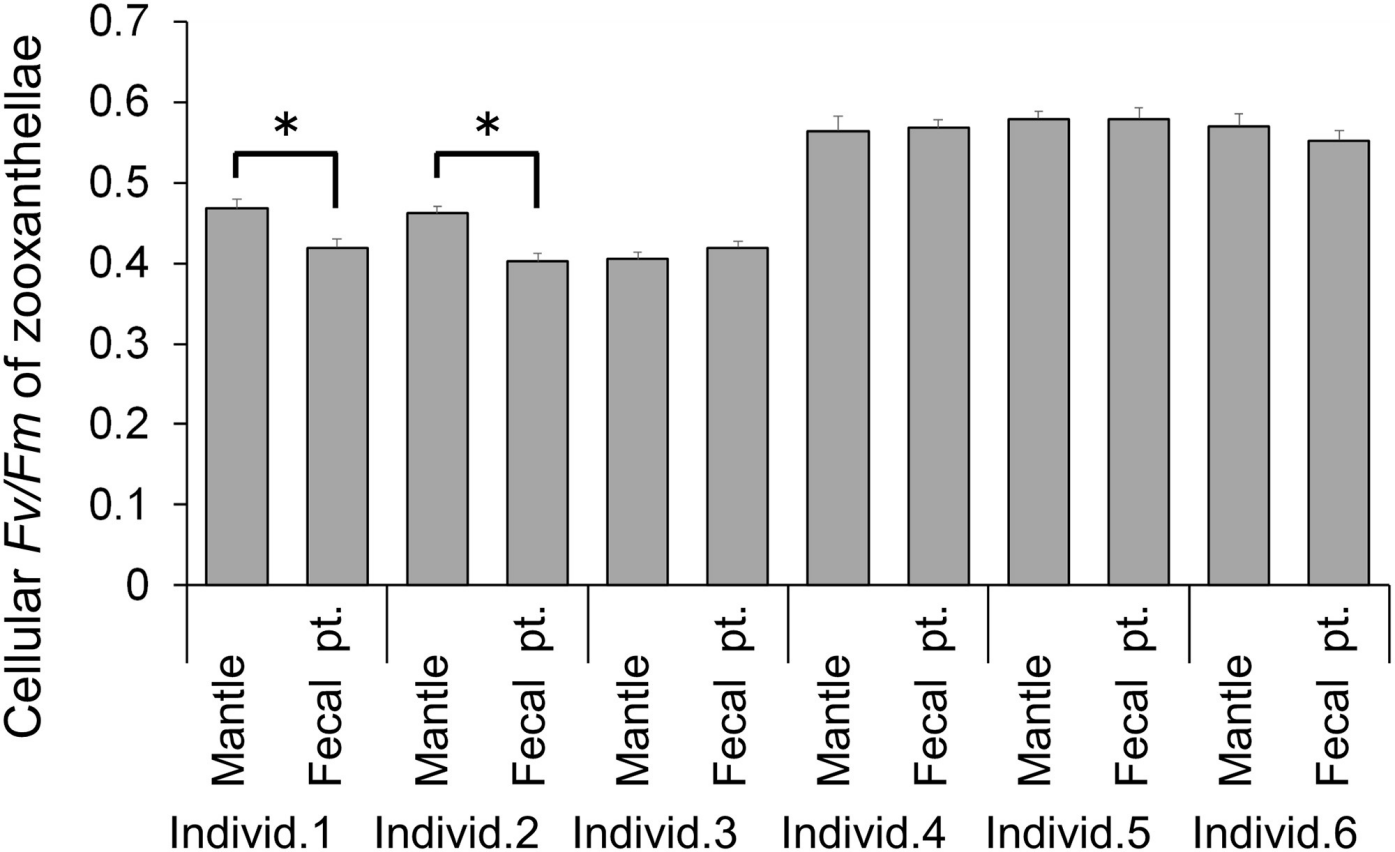
Mean *Fv/Fm* values of the zooxanthellae in the mantle (Mantle) and in fecal pellets (Fecal pt.) of *Tridacna crocea*. Bars indicate standard errors for the measured cells (n = 40 ~ 120). Asterisks indicate significant differences among pairs (*p* < 0.05).

The mean *Fv/Fm* values of zooxanthellae retained in mantles and those in the fecal pellets in each of the six individuals are shown in Fig 4. The average *Fv/Fm* values of each mantle population were 0.41 ± 0.01 (mean ± SE) at minimum (Individual 3) and 0.58 ± 0.01 at maximum (Individual 5). The *Fv/Fm* values of zooxanthellae in the fecal pellets ranged from 0.40 ± 0.01 (Individual 2) to 0.58 ± 0.01 (Individual 5). While significant decreases in the *Fv/Fm* value in the fecal pellets were observed in Individual 1 (df = 204.72, *p* = 3.80 ×10^−3^) and Individual 2 (df = 212.98, *p* = 7.00 × 10^−6^), the decreases were relatively small at 11 and 13% for Individual 1 and Individual 2, respectively. The rest of the individuals maintained similar *Fv/Fm* values in the fecal pellets compared to those in the mantles.

### Proposed process of fecal pellet expulsion

**Fig 5:**
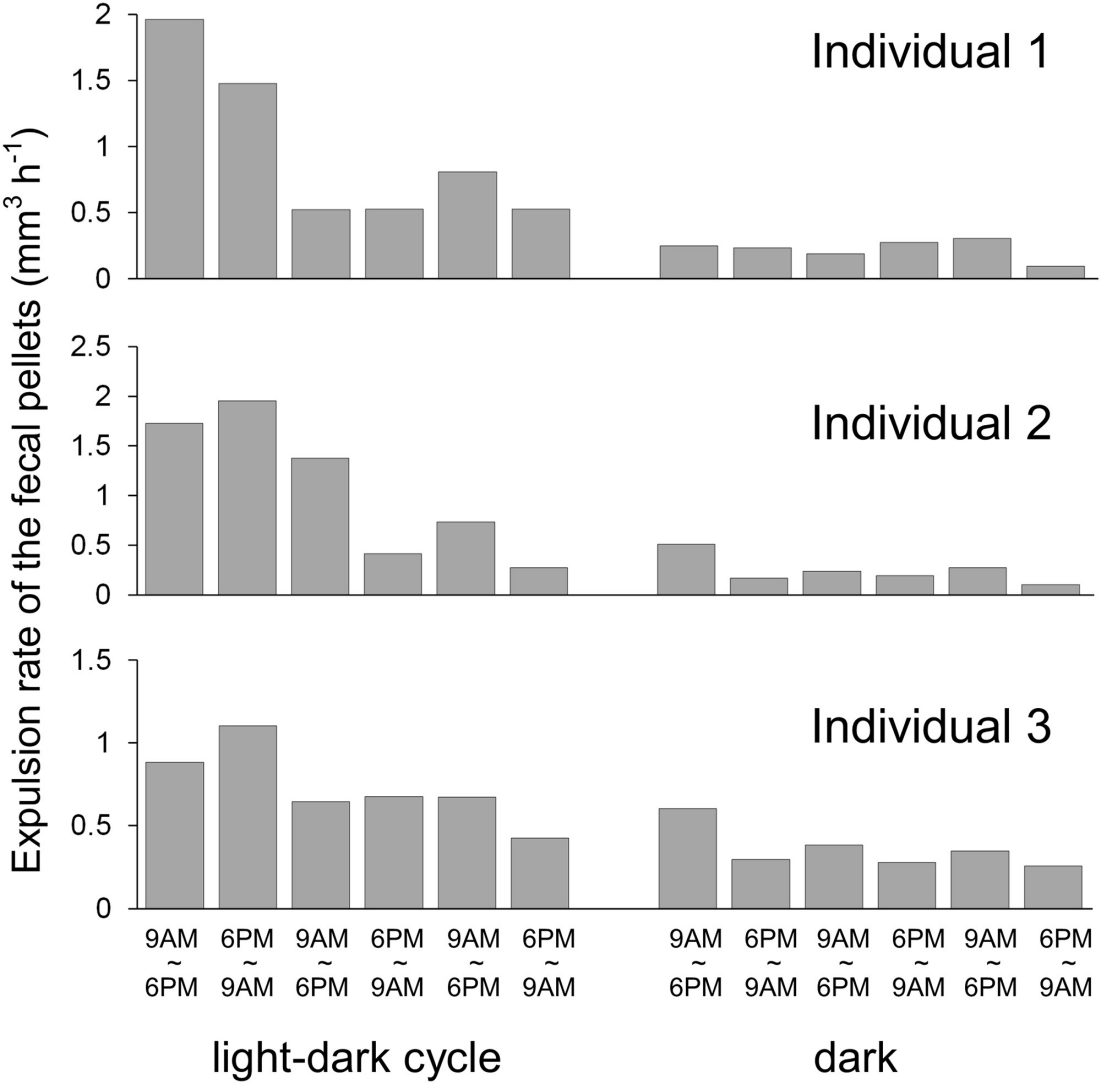
Fecal pellet expulsion rates of three *Tridacna crocea* individuals under a light-dark cycle and continuous dark conditions (dark).

The fecal pellet expulsion rates in the three individuals exposed under a light-dark cycle (12 h light: 12 h dark, continued for 3 days) and continuous dark conditions (24 h dark for 3 days) are shown in Fig 5. Continuous incubation in the small container (a 5 L beaker) might stress the giant clams, and the expulsion rates in Individuals 1 and 2 gradually decreased on the second and third days of the light-dark cycle. Even if such unexpected lowering might occur in the light-dark cycle, the mean expulsion rates (mm^3^ h^-1^) of Individual 1 under the light-dark cycle and dark conditions were 0.97 ± 0.25 (mean ± SE) and 0.22 ± 0.03, respectively. Those of Individual 2 were 1.08 ± 0.29 and 0.25 ± 0.06, and those of Individual 3 were 0.73 ± 0.09 and 0.36 ± 0.05, respectively. In all three individuals, the mean expulsion rate of fecal pellets under the light-dark cycle was significantly higher than that under dark conditions (Individual 1, df = 5.1485, *p* = 0.03; Individual 2, df = 5.3982, *p* = 0.03; Individual 3, df = 7.7557, *p* = 9.00 × 10^−3^).

### Infection ability of the zooxanthellae in the fecal pellets

**Fig 6:**
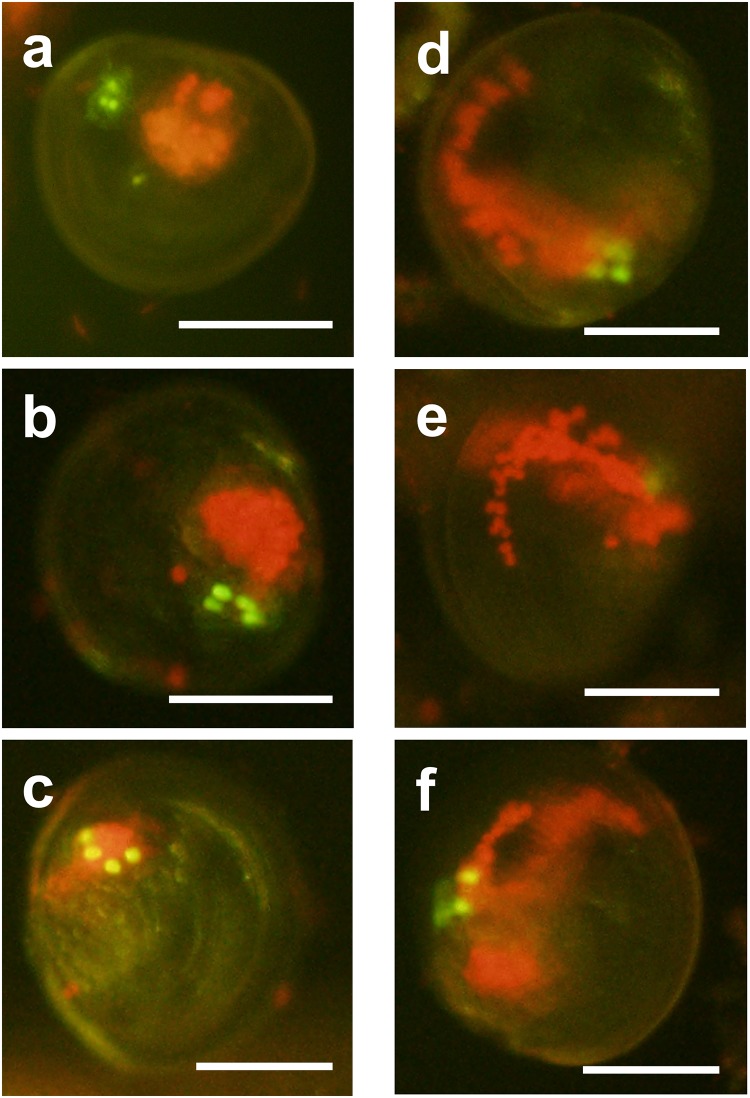
Fluorescence micrographs of *Tridacna squamosa* larvae provided with zooxanthellal sources. (a), (b), (c) Larvae in the uptake stage, showing red chlorophyll fluorescence accumulating within the stomach. (d), (e), (f) Larvae in the symbiosis stage, showing fluorescent granules reaching the mantle edge. Scale bars = 100 μm.

On the 9^th^ day when observation was preliminarily made, larvae with red *in vivo* chlorophyll fluorescence accumulated in the center of a larva (Fig 6a–6c) were dominantly found. They depicted the uptake of zooxanthellae in the stomachs of the larvae (uptake stage). Other larvae with fluorescence particles at the edge (Fig 6d–6f) appeared on the 14^th^ day, indicating the establishment of symbiosis as defined by [15] (symbiosis stage).

**Fig 7:**
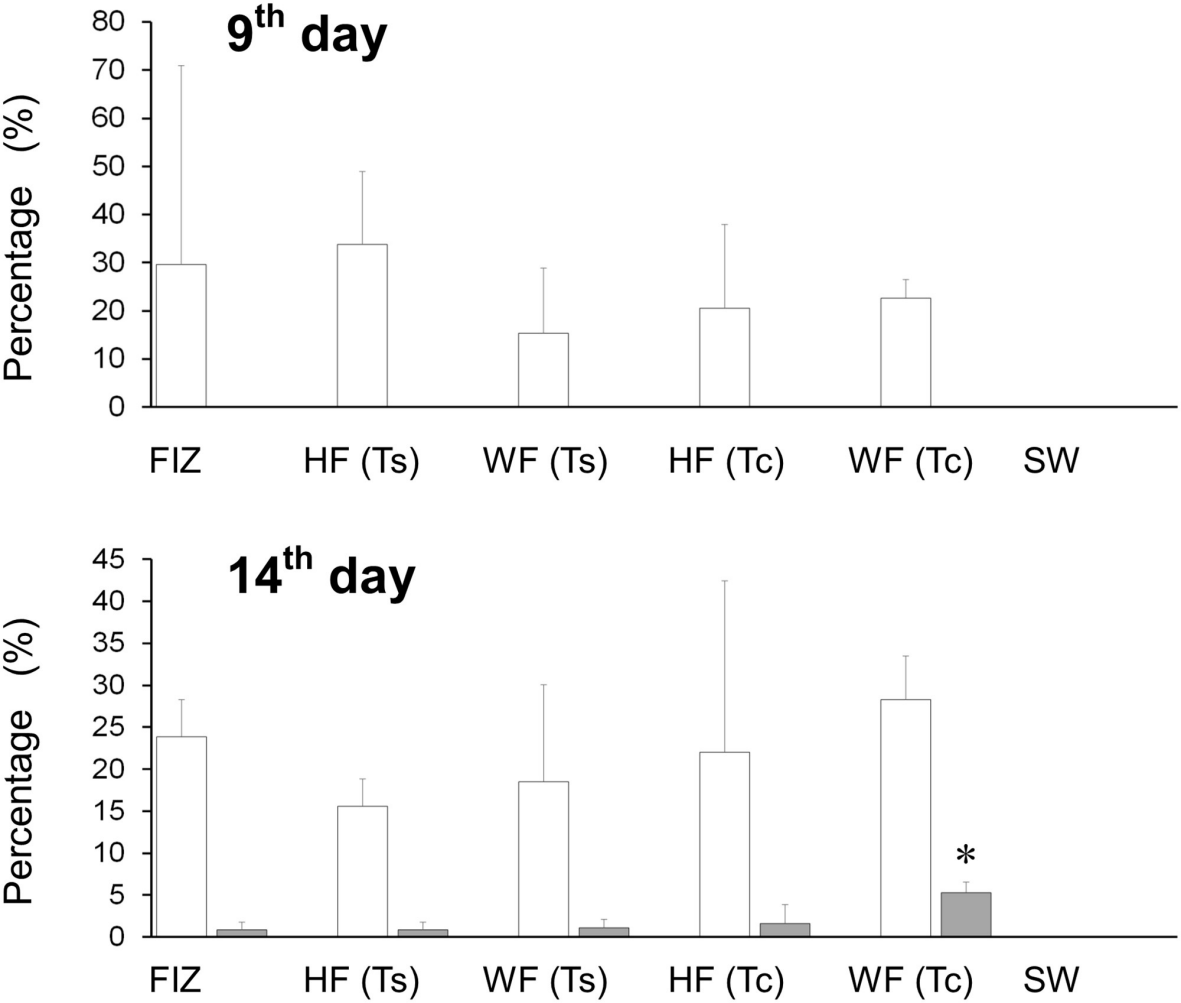
Percentages of *Tridacna squamosa* larvae in the uptake stage (white bars) and in the symbiosis stage (shaded bars) on the 9th day and 14th day after fertilization. FIZ, HF(Ts), WF(Ts), HF (Tc), WF (Tc), and SW indicate freshly isolated zooxanthellae, homogenized fecal pellets of *Tridacna squamosa*, whole intact fecal pellets of *T*. *squamosa*, homogenized fecal pellets of *Tridacna crocea*, whole intact fecal pellets of *T*. *crocea* and a negative control, respectively. Error bars indicate standard deviations based on triplicate experimental units. An asterisk indicates a significant difference from the other treatments (*p* < 0.05, Tukey’s HSD).

The percentages of larvae in the uptake stage and symbiosis stage on each observation day are shown in Fig 7. All the tested zooxanthellal sources were taken up and established symbiotic relationships with *T*. *squamosa* larvae, although the percentage of larvae in the symbiosis stage (shaded bars in Fig 7) was remarkably lower than that in the uptake stage (white bars). In the negative control group in which no zooxanthella cells were provided, the larvae were alive but were not infected by any zooxanthellae (Fig 7, SW), supporting that the zooxanthellae were certainly obtained from the zooxanthellal sources provided during the experimental procedure. On the 9th day, larvae in the symbiosis stage were not observed, and the mean percentages of larvae in the uptake stage averaged across the triplicate beakers of FIZ, HF (Ts), WF (Ts), HF (Tc), WF (Tc), and SW were 29.56 ± 41.44% (numbers of observed individuals = 12, 13 and 34 for each triplicate beaker), 33.86 ± 15.13% (n = 10, 15 and 38), 15.34 ± 13.57% (n = 10, 13 and 19), 20.54 ± 17.46% (n = 10, 13 and 16), 22.61 ± 3.89% (n = 22, 55 and 62), and 0 ± 0% (n = 12, 18 and 21), respectively (mean ± SD). No significant differences were identified among the five zooxanthellal sources (F value = 0.331, df1 = 4, df2 = 10, *p* = 0.851 in one-way ANOVA). On the 14^th^ day, the percentages of larvae in the uptake stage (white bars) averaged across the triplicate beakers of FIZ, HF(Ts), WF (Ts), HF (Tc), WF (Tc), and SW were similar to those on the 9^th^ day or slightly decreased: 23.84 ± 4.48% (n = 112, 114 and 129), 15.57 ± 3.30% (n = 104, 114 and 117), 18.49 ± 11.53% (n = 108, 121 and 130), 22.00 ± 20.46% (n = 102, 121 and 126), 28.33 ± 5.13% (n = 117, 119 and 126), and 0 ± 0% (n = 121, 127 and 135), respectively (mean ± SD). No significant differences were identified among the five zooxanthellal sources (F value = 0.8099, df1 = 4, df2 = 10, *p* = 0.81 in one-way ANOVA). However, on the 14^th^ day, larvae in the symbiosis stage were found, and those percentages (shaded bars) averaged across the triplicate beakers of FIZ, HF(Ts), WF (Ts), HF (Tc), WF (Tc), and SW were 0.88 ± 0.88%, 0.89 ± 0.86%, 1.13 ± 0.99%, 1.64 ± 2.19%, 5.25 ± 1.30%, and 0 ± 0%, respectively (mean ± SD). One-way ANOVA rejected the null hypothesis that there were no differences among the zooxanthellal sources (F value = 5.8233, df1 = 4, df2 = 10, *p* = 0.011). Then, to compare the multiple groups, a Tukey HSD test was performed. The analysis revealed that the larvae provided with zooxanthellal source WF (Tc) showed a significantly higher percentage of individuals in the symbiosis stage than those provided with the other sources (*p* < 0.05, Tukey HSD).

### Zooxanthella identification in the fecal pellets and infected larvae

**Fig 8:**
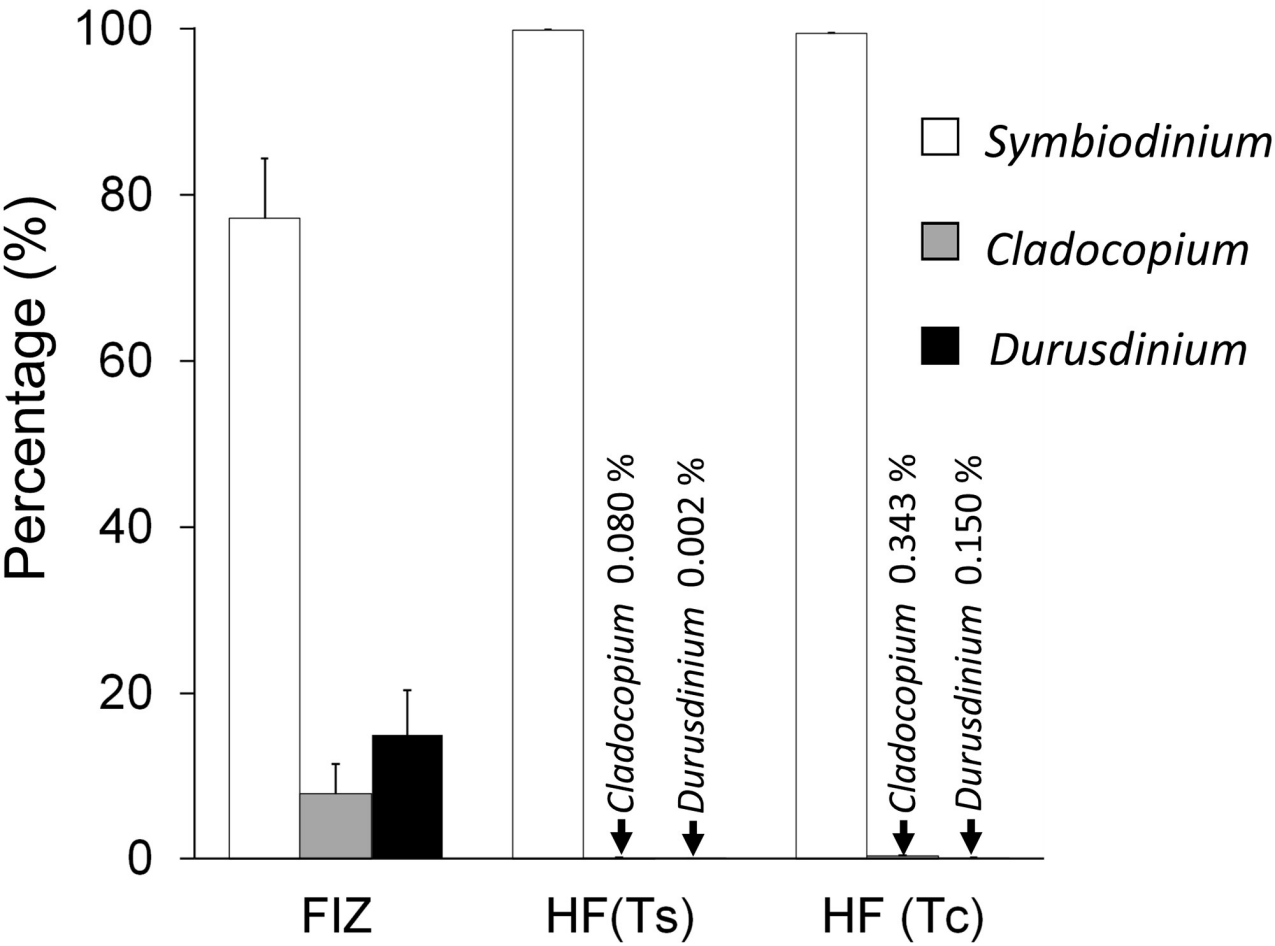
Zooxanthella genus compositions in each zooxanthellal source (averaged across a total of 6 samples). FIZ, HF (Ts), and HF (Tc) indicate freshly isolated zooxanthellae, homogenized fecal pellets of *Tridacna squamosa*, and homogenized fecal pellets of *Tridacna crocea*, respectively. WF(Ts) and WF(Tc) were not analyzed because they were essentially the same as HF(Ts) and HF(Tc). Error bars indicate the standard errors based on six samples.

The genus compositions of the zooxanthellal sources, as determined by quantitative PCR, are shown for each day in S1 Fig and averaged across days in Fig 8. FIZ showed a diverse genus composition; the mean percentages of each genus averaged across six days were 77.15 ± 7.27%, 7.86 ± 3.61%, and 14.98 ± 5.30% (mean ± SE) for *Symbiodinium*, *Cladocopium* and *Durusdinium*, respectively. In contrast, HF(Ts) and HF (Tc) predominantly contained *Symbiodinium* (> 99%) and trace levels of *Cladocopium* and *Durusdinium*. However, the percentages of the latter two genera, 0.002 ~ 0.343%, were calculated based on the significant qPCR signals within the range of standard fitting and thus should not be neglected.

**Fig 9:**
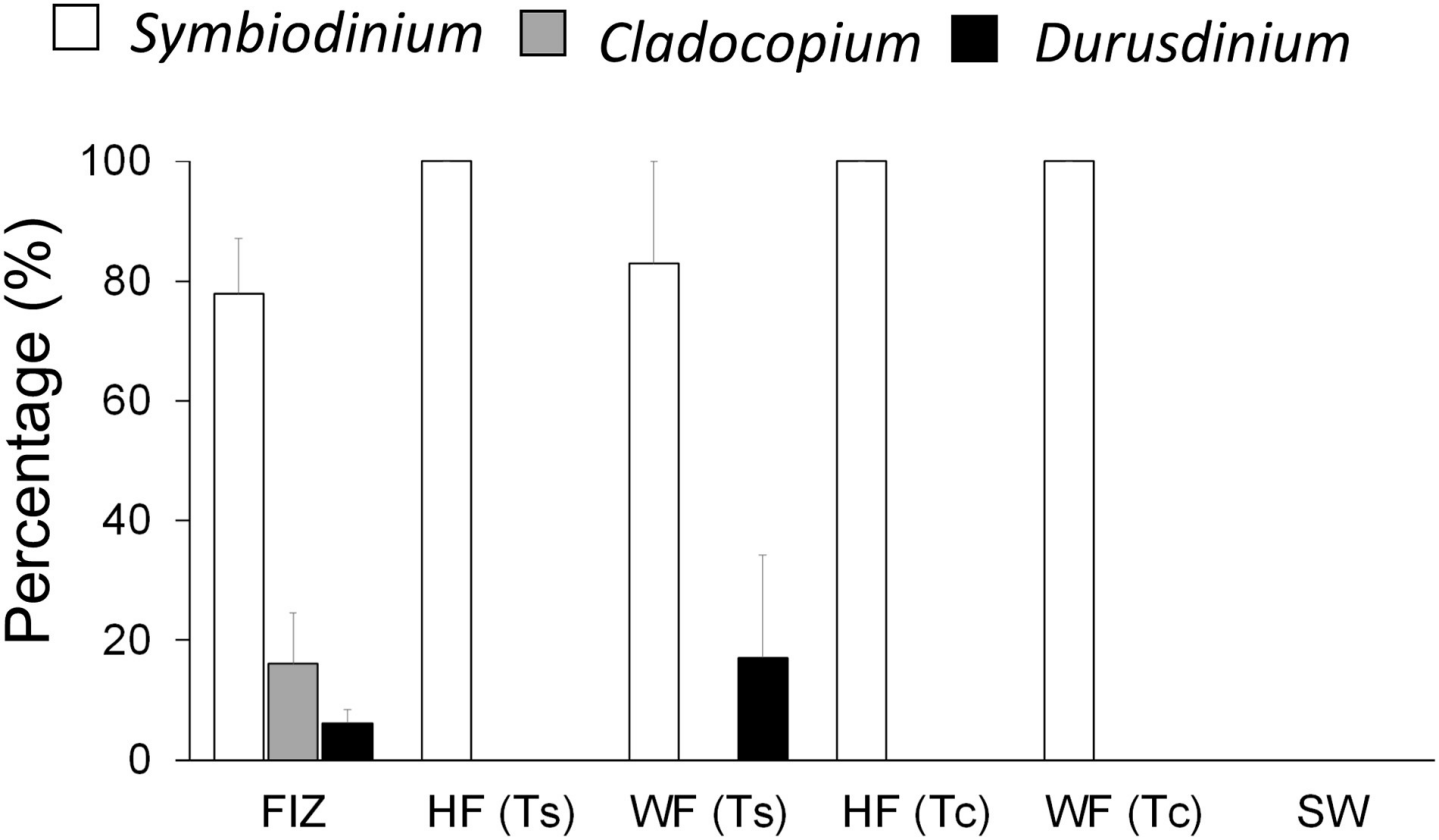
Zooxanthellal genus compositions of the *Tridacna squamosa* larvae incubated with each zooxanthellal source (averaged across triplicate experimental units) 14 days after fertilization. FIZ, HF(Ts), WF(Ts), HF (Tc), WF (Tc), and SW indicate freshly isolated zooxanthellae, homogenized fecal pellets of *Tridacna squamosa*, whole intact fecal pellets of *T*. *squamosa*, homogenized fecal pellets of *Tridacna crocea*, whole intact fecal pellets of *T*. *crocea*, and a negative control, respectively. Error bars indicate standard errors based on triplicate experimental units.

The genus compositions in the larvae on the 14th day are also shown in Fig 9. It is not clear whether these genus compositions were derived during the uptake stage or symbiosis stage, but they should not have been derived from free zooxanthellae but rather from the zooxanthellae taken up by the larvae because of the careful rinsing that occurred at the time of collection. The larvae provided with FIZ showed diverse genera, consisting of S*ymbiodinium*, *Cladocopium* and *Durusdinium*, which was similar to the genera of the zooxanthellal source (FIZ) (Fig 8) but still significantly different (χ^2^ = 6.5187, df = 2, *p* = 0.038). The mean percentages of each genus averaged across the triplicate beakers were 77.82 ± 9.37% of *Symbiodinium*, 16.04 ± 8.49% of *Cladocopium*, and 6.14 ± 2.27% of *Durusdinium* (mean ± SE). In the other experimental treatments, the larvae established symbioses only with *Symbiodinium*, which was consistent with the provided zooxanthellal sources in all cases except the one provided with WF (Ts). The clade composition in the larvae treated with WF (Ts) showed 17.10 ± 17.01% of *Durusdinium*, which occurred because of a significant *Durusdinium* signal obtained for one of the batches.

### Photosynthetic competency of the zooxanthellal sources

**Fig 10:**
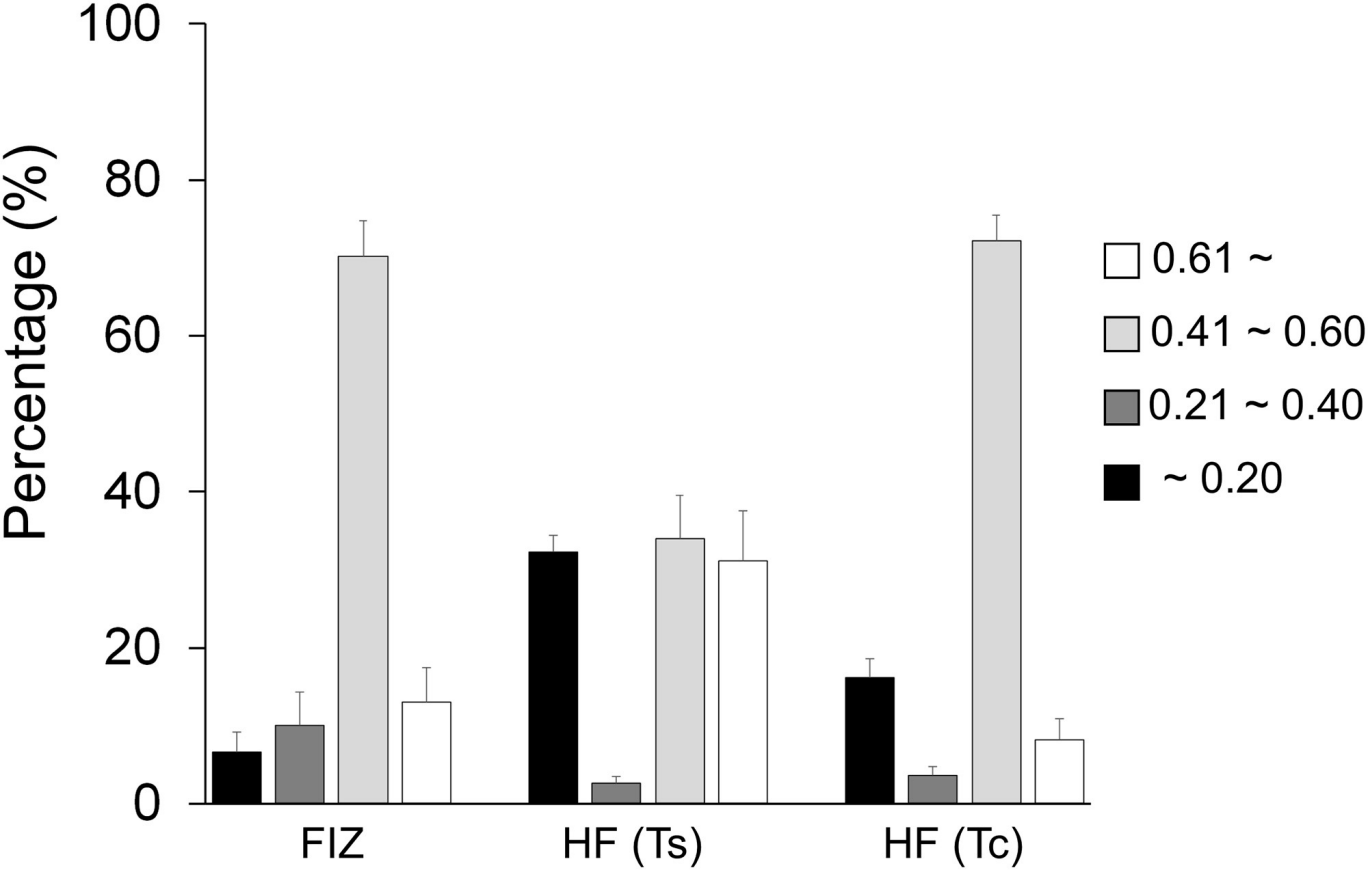
Percentages of the zooxanthella population in each *Fv/Fm* range (averaged over 6 samples). FIZ, HF (Ts), and HF (Tc) indicate freshly isolated zooxanthellae, homogenized fecal pellets of *Tridacna squamosa*, and homogenized fecal pellets of *Tridacna crocea*, respectively. WF(Ts) and WF(Tc) were not analyzed because they were essentially the same as HF(Ts) and HF(Tc). Error bars indicate the standard errors based on 6 samples.

The *Fv/Fm* values of the cells in the zooxanthellal sources, FIZ, HF (Ts) and HF (Tc), were expressed as percentages classified into four categories (i.e., ≤ 0.20, 0.21–0.40, 0.41–0.60, ≥ 0.61) as shown in S2 Fig for daily measurements and Fig 10 for the average. The mean percentages of each *Fv/Fm* range averaged over six days for FIZ, HF (Ts) and HF (Tc) peaked at *Fv/Fm* = 0.41–0.60, and 70.19 ± 4.47%, 33.95 ± 5.53%, and 72.16 ± 3.26% of the populations were within this range for FIZ, HF (Ts) and HF (Tc), respectively. Relatively weak cells, with *Fv/Fm* values below 0.40 [37], accounted for 16.72%, 34.89% and 19.67% of the FIZ, HF (Ts) and HF (Tc), respectively, and the population in the HF (Ts) was more dominated by inactive cells, with *Fv/Fm* values < 0.20 [37].

## Discussion

### Zooxanthella cells in fecal pellets

In all of the individuals, the zooxanthella populations in the fecal pellets were photosynthetically competent, based on the fact that the *Fv/Fm* values were above 0.4, indicating moderate PS II quantum yield [37]. Trench et al. [12] previously reported the photosynthetic competency of zooxanthellae in fecal pellets from *T*. *maxima* by means of ^14^C assimilation, and their finding is now confirmed in *T*. *crocea*. In the present study, the *Fv/Fm* values obtained for the zooxanthellae in the fecal pellets of *T*. *crocea* were comparable to those in the mantle, indicating that these photosynthetically competent zooxanthella cells likely represented excess cells that had grown in the mantle. Our trial to compare expulsion rates between the light-dark cycle and the continuous dark condition may have failed, because there were notable decreases even under the light-dark cycle, which were most likely due to the treatment stress. However, low expulsion rates continuing under dark conditions indicate a decrease in the growing population, further implying that active expulsion might be caused by overflowing of the growing population. However, it has already been suggested that such growing populations are not entirely expelled as competent forms; Maruyama and Heslinga [14] calculated the zooxanthellal growth rates in *T*. *derasa* and showed that 64–89% of newly formed zooxanthellae were missing, implying that some may have been digested by the giant clam. Nevertheless, they also found that 1.6–11.8% of newly formed zooxanthellae appeared in the fecal pellets. We also tried to estimate the percentage of the zooxanthellal population that appeared in the fecal pellets. Although the tested *T*. *crocea* individual was not the one used in the above experimental series, fecal pellets from a *T*. *crocea* individual were retrieved, and their volumes and zooxanthella cell counts were estimated. S3 Fig shows a linear regression between the fecal pellet volume and zooxanthella cell number, and the density was roughly estimated as 2.74 × 10^5^ cells mm^-3^. When applying the volumes of fecal pellets expelled from a giant clam under a light-dark cycle (Fig 5), the average numbers of expelled zooxanthella cells in the fecal pellets of *T*. *crocea* Individuals 1, 2 and 3 were 9.88 × 10^6^, 1.10 × 10^7^, and 7.48 × 10^6^ cells day^-1^, respectively. When considering the total zooxanthella cell number per individual giant clam host, which was based on an assumption regarding the relationship between giant clam size and the number of endosymbiotic zooxanthella cells [38], the total numbers of cells in Individuals 1, 2 and 3 were calculated as 2.66 × 10^8^, 1.85 × 10^8^, and 1.53 × 10^8^ cells, respectively. Accordingly, the percentages of zooxanthellae expelled daily were 3.71%, 5.95%, and 4.89%, respectively, and these values are within the value of 1.6–11.8% reported by [14]. Norton et al. [5] showed that zooxanthellal tubes, which zooxanthellae inhabit and grow within, elongate from the stomach and spread into the mantle area. Therefore, the zooxanthellae in the mantle must pass through the stomach and digestive tract, and consequently the majority of the expelled population could be digested. Nevertheless, a portion of the population did not undergo digestion and maintained intact organelles.

The reason and mechanism by which certain zooxanthellae could escape host digestion remain unclear; however, there has been a number of studies showing that zooxanthella genera or species richness in several giant clam species are influenced by depth, size and geographical locality [19] and by growth-related processes [18]. Selective digestion or avoidance from digestion may render selective retention of some zooxanthella genera in the giant clams.

### Infection ability of the zooxanthellae in the fecal pellets

In this study, all zooxanthellal sources, including those of *T*. *crocea* origins, were taken up and symbiotically established in the *T*. *squamosa* larvae. This study is the first to demonstrate the potential of giant clam fecal pellets as symbiont vectors to giant clam larvae. These results also demonstrate the possibility that fecal pellets are a source of zooxanthellae in coral reefs.

WF (Tc) showed a significantly higher rate of symbiosis than the other treatments, even though it is of heterospecific origin. This observation might have occurred because the fecal pellets of *T*. *crocea* contained more active zooxanthella cells (*Fv/Fm* > 0.4) than those of *T*. *squamosa* (Fig 10). However, the reason why the rate of symbiosis in WF (Tc) even surpassed that in FIZ, which also showed similarly high *Fv/Fm* values, and HF (Tc), which also originated from *T*. *crocea* but was composed of homogenized fecal pellets, remains unclear. The significantly higher rate of symbiosis stage in WF (Tc) may support previous observations in which the rates of uptake and symbiosis in cnidarian larvae (*Anthopleura elegantissima* and *Acropora digitifera* planula larvae) were stimulated by the addition of macerated *Artemia* tissues [39, 40]. Therefore, the whole fecal pellets might induce active uptake by the *T*. *squamosa* larvae, although the animal phyla are completely different. Neo et al. [41] reported that the settlement competency period in *T*. *squamosa* is 14 days, and until then, the larvae actively swim and are able to alter the depth distribution and settlement location. Chemosensory abilities in giant clam larvae could be coupled with their locomotory behavior [42]. If such sensory behavior could control access to preferable zooxanthella sources, a higher rate of symbiosis stage in certain zooxanthellal source may occur but should be addressed in the future. Similarly, a relationship between symbiotic success and exogenous materials was also demonstrated in [43]; the infection rates of four species of tridacnine juveniles provided with zooxanthella cultures significantly decreased when the individuals were reared in 0.2 μm-filtered seawater compared to those reared in natural (nonfiltered) seawater indicating that foreign particles in the natural seawater provided cues to establish the symbiosis.

The *T*. *squamosa* larvae were capable of establishing symbiosis with zooxanthellae in the fecal pellets from a different species of giant clam, which suggests flexibility in the acquisition of symbionts. Kurihara et al. [44] reported that the larvae of *T*. *crocea* and *T*. *maxima* could form symbioses with zooxanthella culture strains isolated from heterospecific clams, and the infection rate was not significantly different from that of a group provided with zooxanthellae isolated from the same species. Mies et al. [45] provided culture strains originating from various animals to *T*. *crocea* larvae and showed that the larvae equally acquired all strains. Our results, however, indicate a significant difference in the abundance ratio of zooxanthellal genera, although it was not obvious, between the ratios of the provided sources and the uptake/symbiosis stage larvae. Even if giant clam larvae can evenly access zooxanthellal sources from different origins, they might select specific zooxanthella genera, similar to the corals. For instance, *Acropora* coral larvae tend to initially take up *Symbiodinium* and/or *Durusdinium*, even though these genera exist at low densities in the environment [27, 46]. Such selective uptake by coral larvae was also observed in laboratory experiments [47]. Because different species of giant clams reared in the same pond showed different preferences for zooxanthella genera [18] and that zooxanthellal genera or species richness in several giant clam species were influenced by environment or locality [19], the selection of suitable zooxanthellae should occur at some growth stage.

Buddemeier and Fautin [48] suggested the adaptive bleaching hypothesis (ABH), that is, environmentally stressed animals change their endosymbiont genera to one that is more suitable to the conditions to which the animal is exposed. The hypothesis is based on two modes: “shuffling”, namely, a shift from background endosymbionts to a major symbiont, or “switching”, namely, the uptake of external zooxanthellae. The latter mode has been reported to occur in anemones [49] and soft corals [50]. Lim et al. [19] reported zooxanthella genera or species richness in several giant clam species were influenced by depth, size and geographical locality. DeBoer et al. [17] investigated the zooxanthellae cladal compositions of three giant clam species in Indonesia and revealed that those inhabiting warmer areas harbored *Cladocopium* and *Durusdinium*, whereas clams hosting *Symbiodinium* occurred in cooler waters. Additionally, they found zooxanthellal type D1a, known as a “host generalist” (e.g., [51]), in the three species, and it was detected in higher numbers in the giant clams inhabiting warmer waters than those in cooler waters. It has been reported that some members of *Durusdinium*, including type D1a, show high thermal tolerance (e.g., [52, 53]). Considering the assumed process by which fecal pellets are formed and expelled, it is plausible to think that under high- temperature conditions, more thermal-tolerant genera or types are more likely to be expelled in the fecal pellets and more accessible for the giant clam juveniles. This possibility is likely because the zooxanthellae in the fecal pellets were excess individuals from the mantle that developed under the environmental conditions to which the clam was exposed. If this is true, this mechanism might enhance the environmental resilience of the juveniles and should be further investigated.

## Conclusion

In the present study, we demonstrated that zooxanthella populations in the fecal pellets of *T*. *crocea* were photosynthetically competent and had intact ultrastructures. These populations probably overflowed from the mantle and escaped from digestion in the stomach of the giant clam. Additionally, we examined the ability of these zooxanthella populations to infect *Tridacna* s*quamosa* larvae. The larvae acquired zooxanthellae from the fecal pellets expelled from both *T*. *squamosa* and *T*. *crocea*, and the rate at which the symbiosis stage was reached was highest in the larvae given whole (nonhomogenized) fecal pellets of *T*. *crocea*. Not obvious but significant differences in the zooxanthella genera provided from the fecal pellets and retained in the larvae were observed. These results suggest that *T*. *squamosa* larvae were capable of acquiring zooxanthellae, preferably via fecal pellets, even from different species of giant clam, and may selectively establish symbiosis with specific zooxanthella genera. Considering the assumed process by which a part of the overflowed population of zooxanthella that grew in the mantle was expelled and inherited to the new generation of giant clams, this mechanism might increase the possibility for the juvenile to access more “environmentally suitable” zooxanthella genera or species, and if this is true, this mechanism might enhance the environmental resilience of the juveniles. This finding, as well as the possibility that the fecal pellets that might transport zooxanthellae to other animals (e.g., corals), should be further investigated.

## Supporting information

S1 Fig Zooxanthella clade compositions of each zooxanthellal source (on each day) for each supply batch. FIZ, HF (Ts), and HF (Tc) indicate freshly isolated zooxanthellae, homogenized fecal pellets of *Tridacna squamosa*, and homogenized fecal pellets of *Tridacna crocea*, respectively.
(TIF)

S2 Fig Percentage of the zooxanthella populations in each *Fv/Fm* category (on each day). FIZ, HF (Ts), and HF (Tc) indicate freshly isolated zooxanthellae, homogenized fecal pellets of *Tridacna squamosa*, and homogenized fecal pellets of *Tridacna crocea*, respectively.
(TIF)

S3 Fig Relationship between fecal pellet volume and zooxanthella cell number. Supporting data. xlsx.
(TIF)

S1 Supporting data Data used for Figs 4, 5, 7, 8, 9 and 10. (XLSX)
