# Supplementary figures and images for: Study on expelled but viable zooxanthellae from giant clams, with an emphasis on their potential as subsequent symbiont sources
Source: PLoS One. 2019 Jul 19;14(7):e0220141. doi: 10.1371/journal.pone.0220141 (PMC6641532; doi:10.1371/journal.pone.0220141)

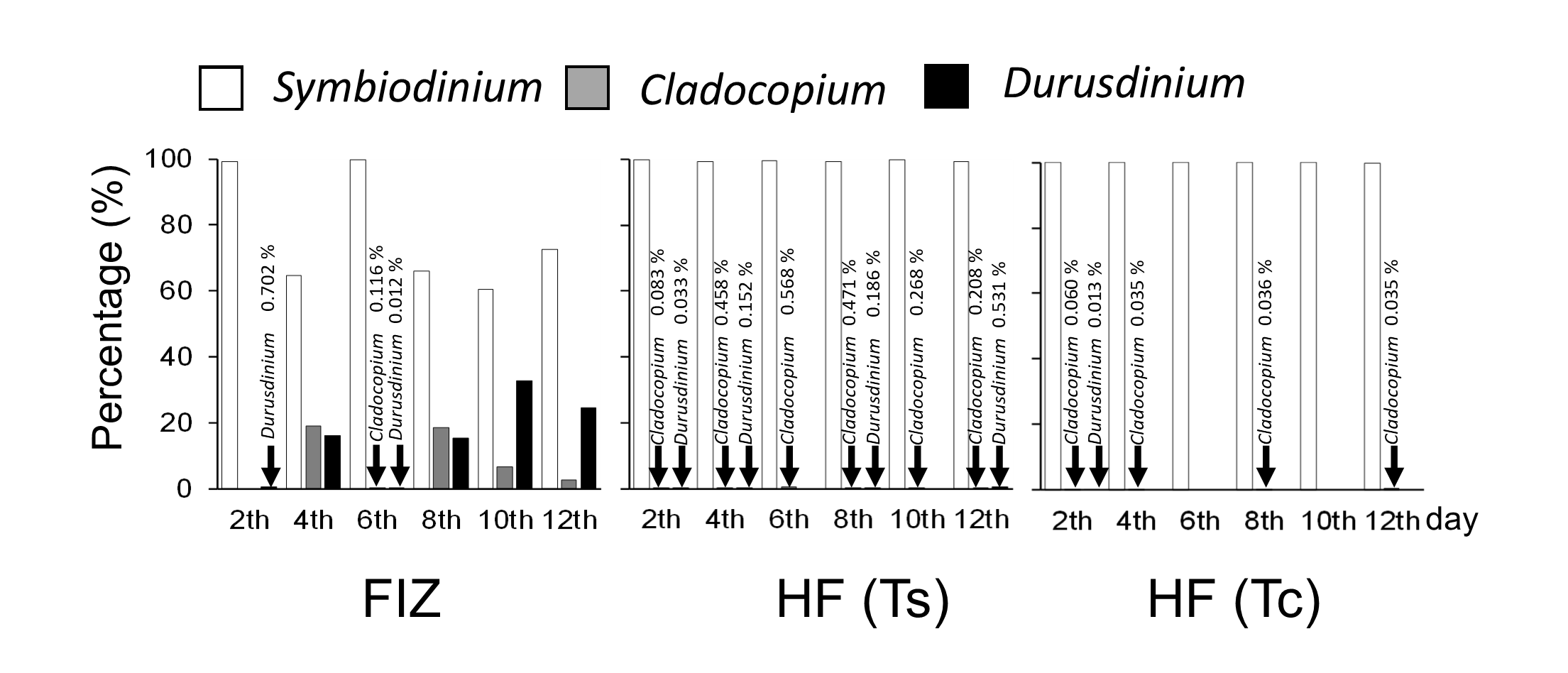

Supplement: S1 Fig — FIZ, HF (Ts), and HF (Tc) indicate freshly isolated zooxanthellae, homogenized fecal pellets of Tridacna squamosa, and homogenized fecal pellets of Tridacna crocea, respectively. (TIF) [file pone.0220141.s001.tif]

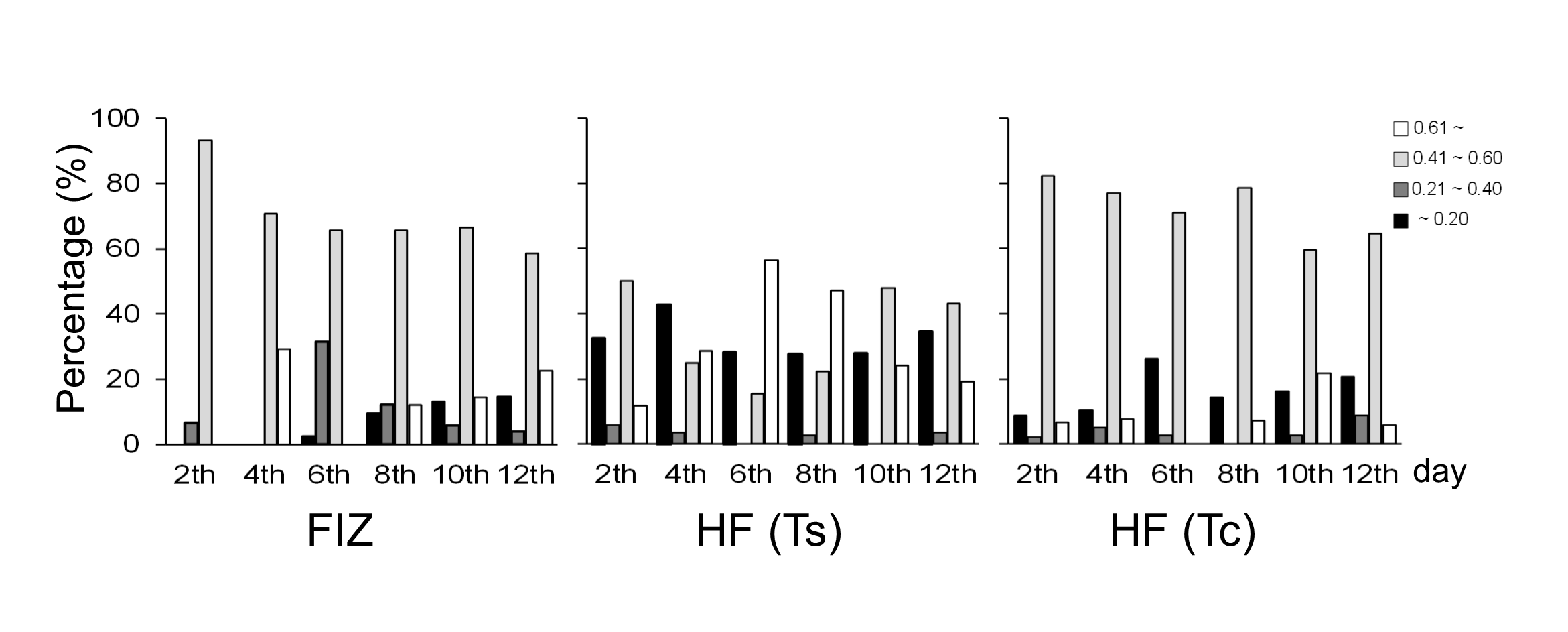

Supplement: S2 Fig — FIZ, HF (Ts), and HF (Tc) indicate freshly isolated zooxanthellae, homogenized fecal pellets of Tridacna squamosa, and homogenized fecal pellets of Tridacna crocea, respectively. (TIF) [file pone.0220141.s002.tif]

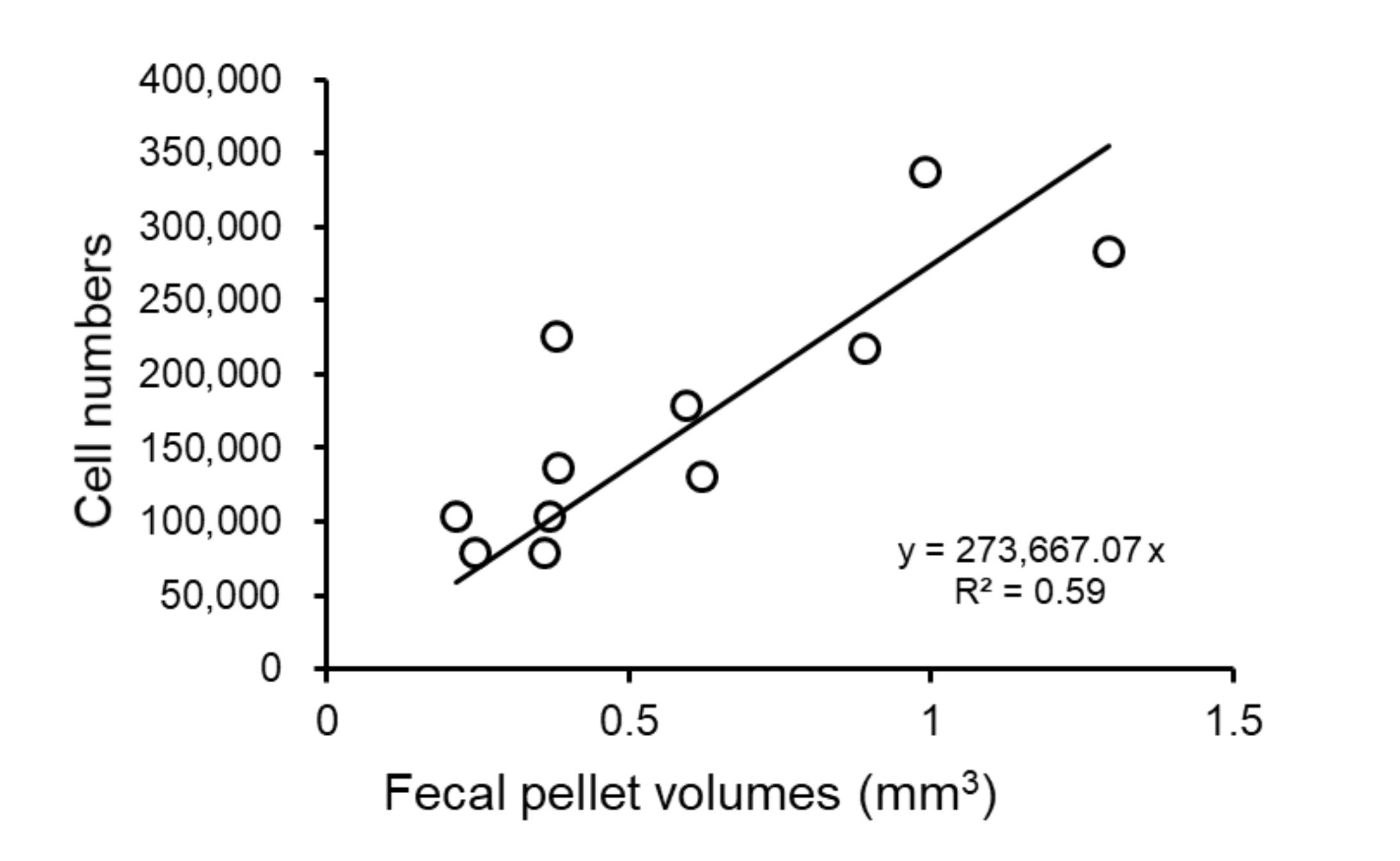

Supplement: S3 Fig — Supporting data. xlsx. (TIF) [file pone.0220141.s003.tif]
